# Neutrophil elastase aggravates periodontitis by disrupting gingival epithelial barrier via cleaving cell adhesion molecules

**DOI:** 10.1038/s41598-022-12358-3

**Published:** 2022-05-17

**Authors:** Takumi Hiyoshi, Hisanori Domon, Tomoki Maekawa, Hikaru Tamura, Toshihito Isono, Satoru Hirayama, Karin Sasagawa, Fumio Takizawa, Koichi Tabeta, Yutaka Terao

**Affiliations:** 1 Division of Microbiology and Infectious Diseases, Niigata University Graduate School of Medical and Dental Sciences, 2-5274, Gakkocho-dori, Chuo-ku, Niigata-shi, Niigata, 951-8514 Japan; 2 Division of Periodontology, Niigata University Graduate School of Medical and Dental Sciences, Niigata, Japan; 3 Center for Advanced Oral Science, Niigata University Graduate School of Medical and Dental Sciences, Niigata, Japan

**Keywords:** Periodontitis, Bacterial infection, Cell adhesion, Cytokines, Inflammation

## Abstract

Neutrophil elastase (NE) functions as a host defense factor; however, excessive NE activity can potentially destroy human tissues. Although NE activity is positively correlated to gingival crevicular fluid and clinical attachment loss in periodontitis, the underlying mechanisms by which NE aggravates periodontitis remain elusive. In this study, we investigated how NE induces periodontitis severity and whether NE inhibitors were efficacious in periodontitis treatment. In a ligature-induced murine model of periodontitis, neutrophil recruitment, NE activity, and periodontal bone loss were increased in the periodontal tissue. Local administration of an NE inhibitor significantly decreased NE activity in periodontal tissue and attenuated periodontal bone loss. Furthermore, the transcription of proinflammatory cytokines in the gingiva, which was significantly upregulated in the model of periodontitis, was significantly downregulated by NE inhibitor injection. An in vitro study demonstrated that NE cleaved cell adhesion molecules, such as desmoglein 1, occludin, and E-cadherin, and induced exfoliation of the epithelial keratinous layer in three-dimensional human oral epithelial tissue models. The permeability of fluorescein-5-isothiocyanate-dextran or periodontal pathogen was significantly increased by NE treatment in the human gingival epithelial monolayer. These findings suggest that NE induces the disruption of the gingival epithelial barrier and bacterial invasion in periodontal tissues, aggravating periodontitis.

## Introduction

Periodontitis is a bacteria-induced inflammatory disease that leads to tooth loss. The etiology of this disease is the bacterial load in the human oral cavity. However, it is clear that the host inflammatory response to microbial challenge causes periodontal tissue destruction^1^. The ideal host response is that microbial stress is eliminated and the tissue returns to a homeostatic state, which is defined by an absence of inflammation. If the resolution process of inflammation fails, a tissue-injurious inflammatory state is triggered^2^. Since the goal of interventions is the return of tissue to homeostasis^3^, therapeutic strategies targeting inflammation are essential to treat periodontitis.

The initial response to acute periodontal inflammation is the physiological response to the oral microbial challenge to recruit leukocytes to sites of infection^3^. Neutrophils are required for homeostatic immunity and contribute to a clinically healthy periodontal tissue; however, supernumerary migration of neutrophils to periodontal tissue can cause tissue damage through the release of inflammatory molecules and tissue‐degrading proteases in periodontitis^4^.

Neutrophil elastase (NE) is an abundant proteinase stored in the azurophilic granules of neutrophils and is an indicator of neutrophil activity. NE degrades microbiological components in conjunction with phagocytosis, acting as a host defense mechanism^5^. NE works immunologically only in the cells of neutrophils, whereas excessive NE activity damages host tissues by proteolytic function. Inflammatory lung diseases are characterized by uncontrolled high NE levels, and NE inhibitors have shown some clinical benefits and reduce inflammation^6^. In experimental gingivitis in humans, NE levels were increased in the gingival crevicular fluid, and removal of dental plaque decreased the enzyme levels^7^. Additionally, patients with periodontitis showed a positive correlation between NE activity in the gingival crevicular fluid and clinical attachment loss^8^. Our previous study revealed that NE caused detachment and death of human gingival cells after leukotoxin-dependent neutrophil death and NE leakage from inside dead neutrophils^9^.

In this study, we hypothesized that high levels of NE activity caused by an inflammatory response against oral bacteria aggravates periodontitis. We examined the NE activity in periodontal tissue in a ligation-induced murine model of periodontitis. Moreover, we investigated the effects of NE inhibitors on alveolar bone loss, bacterial load, and proinflammatory cytokine gene transcription in the gingiva in a murine model. Furthermore, we evaluated whether NE cleaves cell adhesion molecules and induces the disruption of the gingival epithelial barrier and expansion of bacterial infection in human oral epithelial tissue models in vitro.

## Results

### Recruitment of neutrophils results in the increase of NE activity in the inflamed gingiva

**Figure 1:**
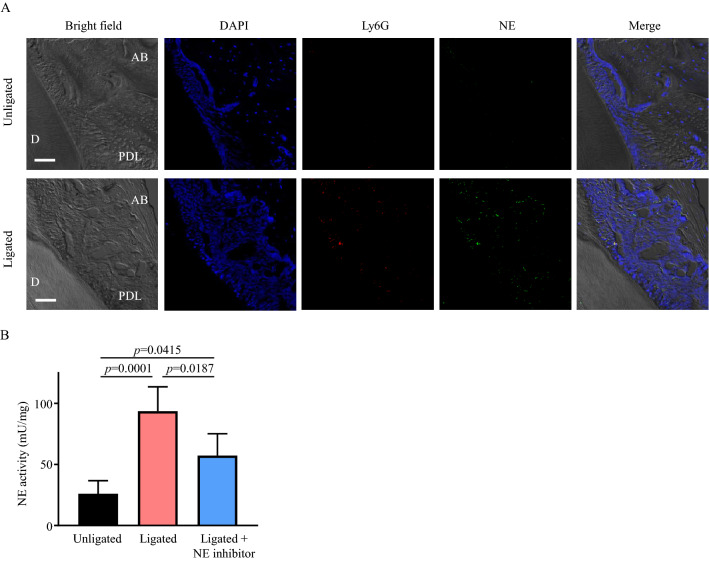
Neutrophil elastase activity is increased in periodontal tissue by ligature-induced periodontitis. (**A**, **B**) PBS or NE inhibitor (50 μg in 5 μL) was injected into the palatal gingiva of a ligature-induced murine model of periodontitis once a day for 7 (**A**) or 3 (**B**) days. (**A**) Frozen maxillae sections (10 μm) were stained with anti-NE antibody (green), anti-Ly6G antibody (red), and DAPI (nucleus; blue). Representative fluorescence images observed using a confocal laser scanning microscope are shown. Scale bar: 50 μm. (**B**) NE activity in supernatants from the homogenized palatal gingiva was evaluated. Data are presented as the mean ± SD (n = 5 per group). The group means were compared using one-way analysis of variance with Tukey’s multiple comparison test. *AB* alveolar bone, *D* dentine, *DAPI* 4′,6-diamidino-2-phenylindole, *Ly6G* lymphocyte antigen 6 complex locus G, *NE* neutrophil elastase, *PDL* periodontal ligament, *SD* standard deviation.

NE activity in the gingival crevicular fluid is positively correlated with clinical attachment loss^8^. Therefore, we first analyzed the localization of neutrophils and NE in a ligature-induced murine model of periodontitis. As shown in Fig. 1A, lymphocyte antigen 6 complex locus G (Ly6G; neutrophils, red) and NE (green) in the periodontal ligament of the ligated group were higher than those in the unligated group. Additionally, NE activity in the gingival tissue was significantly elevated by tooth ligation (Fig. 1B). Furthermore, NE activity was significantly lower in the NE inhibitor-injected group than in the uninjected group (Fig. 1B).

### NE inhibitor attenuates the periodontal bone loss induced by tooth ligation

**Figure 2:**
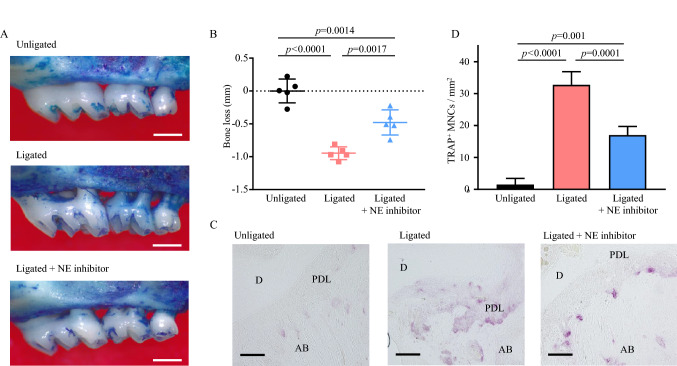
Neutrophil elastase inhibitor inhibits ligature-induced alveolar bone loss. (**A**) Representative images of the maxillae from indicated treatment groups 8 days after ligature placement. Scale bar: 0.5 mm. (**B**) Periodontal bone loss was assessed using a stereoscopic microscope. Negative values indicate bone loss relative to the unligated control. (**C**, **D**) Frozen maxillae sections were stained with tartrate-resistant acid phosphatase. (**C**) Representative images obtained by optical microscopy are shown. Scale bar: 50 μm. (**D**) All the TRAP-positive multinucleated giant cells in the periodontal ligament of ligated second molar were counted in five random coronal sections. Data are presented as the mean ± SD (n = 5 per group). The group means were compared using one-way analysis of variance with Tukey’s multiple comparison test. *AB* alveolar bone, *D* dentine, *MNCs* multinucleated cells, *NE* neutrophil elastase, *PDL* periodontal ligament, *SD* standard deviation, *TRAP* tartrate-resistant acid phosphatase.

We next investigated the protective effect of the NE inhibitor against periodontal bone loss. Figure 2A and B shows that tooth ligation induced significant bone loss compared with the unligated group, and NE inhibition significantly attenuated bone loss compared with the ligated group. The number of tartrate-resistant acid phosphatase (TRAP)-positive osteoclasts on the surface of the alveolar bone in the ligated group was also higher than that in mice from the unligated group (Fig. 2C and D). The injection of the NE inhibitor significantly reduced the number of osteoclasts in the bone tissue sections, which was in agreement with the bone loss measurements.

### NE inhibitor downregulates the transcription of proinflammatory cytokine gene induced by tooth ligation

**Figure 3:**
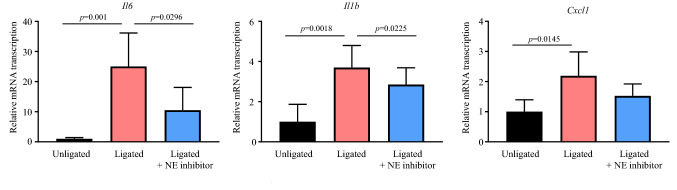
Neutrophil elastase inhibitor decreases the gene transcription of proinflammatory cytokines in the murine gingiva. Messenger RNA transcription levels of *Il6*, *Il1b*, and *Cxcl1* in the palatal gingiva of mice from the indicated groups were determined by real-time PCR. Relative mRNA levels were normalized to those of *Gapdh*. Data are presented as the mean ± SD (n = 5 per group). The group means were compared using one-way analysis of variance with Tukey’s multiple comparison test. *NE* neutrophil elastase, *SD* standard deviation.

Excessive host immunity against microbes is an essential factor in the progression of periodontal disease^10^. Therefore, we next investigated whether injection of an NE inhibitor modulates the transcription of proinflammatory cytokines in the gingiva. Figure 3 shows that the mRNA transcription of *Il6*, *Il1b*, and C*xcl1* significantly increased in the gingiva of the ligated group compared with that in the gingiva of the unligated group. Additionally, the transcription of *Il6* and *Il1b* was significantly downregulated in the gingiva of the NE inhibitor-injected group compared with that in the uninjected group. In contrast, the injection of NE inhibitor induced no significant difference in total bacterial load in the ligature under both aerobic and anaerobic conditions (Supplemental Fig. S1). These findings suggest that NE inhibitors downregulate *Il6* and *Il1b* cytokines without affecting the bacterial number.

### NE cleaves cell adhesion molecules and induces exfoliation of the epithelial keratinous layer

**Figure 4:**
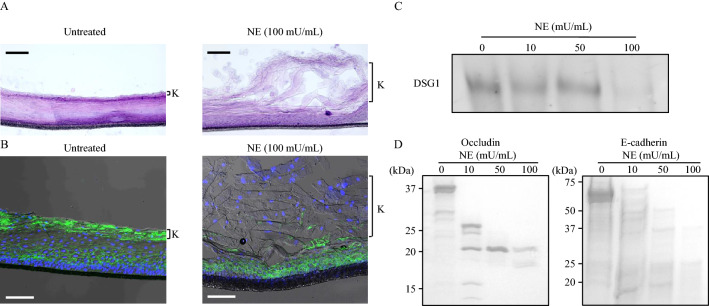
Neutrophil elastase induces exfoliation of gingival epithelial keratinous layer via cleavage of cell adhesion molecule. (**A**, **B**) Three-dimensional human oral epithelial tissue models were exposed to 100 mU/mL NE followed by 12-h incubation. (**A**) Frozen sections were stained with hematoxylin and eosin. Representative images obtained by optical microscopy are shown. Scale bar: 100 μm. (**B**) Representative fluorescence microscopy images of the sections stained for DAPI (nucleus, blue) and DSG1 (green) are shown. Scale bar: 100 μm. K in Fig. 5A and B indicates keratinous layer. (**C**, **D**) Recombinant human DSG1, occludin, and E-cadherin were exposed to NE (10, 50, and 100 mU/mL) for 3 h and determined by western blotting (**C**) or Coomassie Brilliant Blue staining (**D**). *DSG1* desmoglein 1, *NE* neutrophil elastase.

Gingival epithelial cells play a crucial role in the progression of periodontitis by acting as a physical barrier between the outside environment and host^11^. However, NE has been reported to induce detrimental effects on the human nasal mucosal barrier with increased permeability, allowing for potential bacterial invasion^12^. Therefore, we investigated whether NE induces the disruption of periodontal epithelial tissue using three-dimensional human oral epithelial tissue models in vitro. As shown in Fig. 4A, NE induced exfoliation of the gingival epithelial keratinous layer with cell–cell adhesion disruption. Furthermore, NE decreased the expression level of desmoglein 1 (DSG1), a cell adhesion molecule, on the exfoliated epithelial keratinous layer compared with the unligated group (Fig. 4B). Figure 4C shows that recombinant DSG1 was cleaved by 100 mU/mL of NE. Furthermore, NE also cleaved other cell adhesion molecules, occludin, and E-cadherin (Fig. 4D).

### NE disrupts the periodontal epithelial barrier

**Figure 5:**
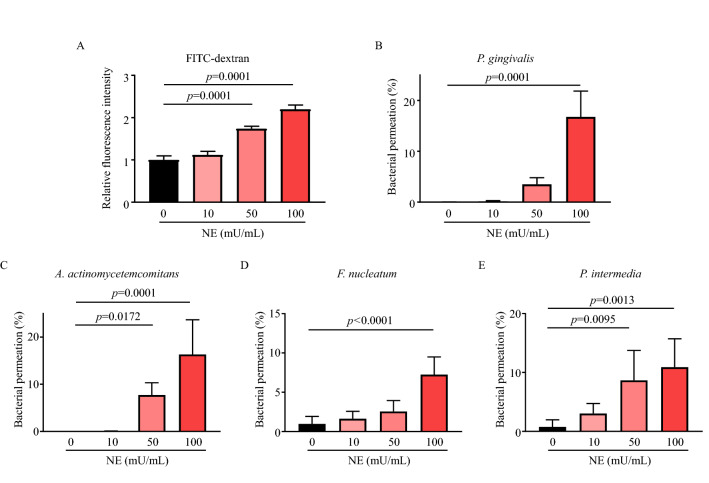
Neutrophil elastase induces disruption of periodontal epithelial barrier. (**A**–**E**) Monolayers of human gingival epithelial cells were exposed to 10–100 mU/mL NE for 3 h. (**A**) Transepithelial permeability was determined by measuring 70 kDa of FITC-dextran fluorescence intensity in the medium from the lower chamber compartments. (**B**–**E**) The transepithelial permeability of each bacterium was determined using the colony counting method. Data are presented as the mean ± SD (n = 5 per group). The group means were compared using one-way analysis of variance with Dunnett’s multiple comparison test. *NE* neutrophil elastase.

To determine whether NE disrupts the epithelial barrier, we performed an in vitro permeability assay using fluorescein-5-isothiocyanate (FITC)-dextran or periodontal pathogen. NE treatment significantly increased FITC-dextran permeation across the Ca9-22 cell monolayer in a dose-dependent manner (Fig. 5A). Additionally, NE treatment enhanced bacterial invasion across the Ca9-22 cell monolayer (Fig. 5B–E). Supplemental Figure S2 shows that NE treatment did not induce a significant decrease in bacterial viability against periodontal pathogen used in this study at concentrations of up to 100 mU/mL, except for *F. nucleatum*.

## Discussion

This study revealed that neutrophil recruitment and NE activity in inflamed periodontal tissue increased in a ligation-induced murine model of periodontitis. Additionally, NE inhibition decreased alveolar bone loss and transcription of proinflammatory cytokine genes but did not affect the bacterial load in ligatures. An in vitro study suggested that NE disrupts the gingival epithelial barrier by degrading cell adhesion molecules, allowing periodontal pathogen to penetrate the periodontal tissues. These findings suggest that the disruptive effect of NE on the gingival epithelial barrier aggravates periodontitis via the expansion of bacterial infection.

Neutrophils are major effectors of infection defense and play an important role in tissue injury. In periodontitis, a large number of neutrophils infiltrate the gingival tissue and sulcus and can eliminate a wide variety of bacteria via multiple mechanisms^2,13^. However, it has been reported that the number of neutrophils correlates positively with periodontitis severity^14^. Furthermore, clinical evidence indicates that neutrophils are responsible for a substantial portion of the inflammatory tissue destruction^15^. Consistent with these studies, neutrophil levels in the periodontal ligament were increased in a ligature-induced murine model of periodontitis with significant periodontal bone loss in the present study. The positive/negative effects of neutrophils on periodontitis have not been fully elucidated; thus, the complicated role of neutrophils in periodontitis should be investigated further.

Excessive extracellular NE, released from neutrophils by bacteria and their products, induces host tissue damage and periodontal bone destruction^16^. Accordingly, NE activity must be tightly regulated by blood plasma‐derived and tissue-derived protease inhibitors to maintain homeostasis. In the gingiva without inflammation, intrinsic antiprotease capacity overwhelms NE activity^17^. However, in patients with periodontitis, NE activity is increased in the gingival crevicular fluid^18^. One reason for this is that some periodontal pathogens stimulate the release of NE while escaping from host‐derived proteases by producing protease inhibitors. The outer membrane component of *Treponema denticola* can trigger protease release from neutrophil azurophilic granules^19^. Additionally, *P. gingivalis* degrades elastase-specific protease inhibitors, such as elafin, and secretory leukocyte peptidase inhibitors, by producing proteolytic enzymes^20^. A clinical study also reported that NE activity in the gingival crevicular fluid was positively associated with the counts of periodontal pathogen, including *P. gingivalis*, *A. actinomycetemcomitans*, *Tannerella forsythia*, and *T. denticola*^21^. Furthermore, this study revealed that NE activity, inducing periodontal tissue damage, exhibited minimum bactericidal activity against periodontal pathogen. These findings suggest that extracellular NE is exploited by periodontal pathogen as an etiological agent, rather than NE, which controls infection in periodontitis.

Sivelestat is an NE inhibitor that is selective, reversible, and competitive against NE. The NE inhibitor has been approved clinically for the treatment of patients with acute lung injury associated with systemic inflammatory response syndrome and is useful in such patients^22^. The NE inhibitor also showed clinical benefit in acute respiratory distress syndrome characterized by massive infiltration of neutrophils, monocytes, and lymphocytes^23^. These studies suggest that NE inhibition could be a potential treatment for inflammatory diseases with neutrophil recruitment, including periodontitis. Additionally, considering an increase in antimicrobial-resistant bacteria, which is a major global public health problem, reducing the consumption of antimicrobials and developing alternatives to antimicrobials used in periodontitis is necessary^24^. In this study, the inhibition of NE activity in periodontal tissue suppressed the transcription of proinflammatory cytokine genes and alveolar bone loss. Despite limitations of animal-based models in predicting human responses and the challenges in clinical application, NE inhibitors could be applied as a novel and non-antibiotic therapeutic treatment in periodontitis.

Cell adhesion molecules play key roles in stabilizing the gingival epithelial barrier and bacterial invasion^25,26^. It has been reported that *P. gingivalis* gingipains disrupt the barrier function of stratified squamous epithelium via degradation of cell adhesion molecules, allowing bacterial virulence factors to penetrate subepithelial tissues^27^. Additionally, *A. actinomycetemcomitans* also disrupts the gingival epithelial barrier by cytolethal distending toxin inducing the subsequent penetration of exogenous pathogens into the host tissues^28^. Penetrated bacterial products induce degradation of the connective tissue and bone, damaging the supporting structures of the teeth. Therefore, early disruption of the epithelial barrier in the development of periodontal disease has significant consequences for oral health^29^. When the bacterial load is low, neutrophils act as a barrier within the gingival crevice, preventing the passage of bacteria deeper into the subjacent gingival connective tissue^16^. However, excessive neutrophils have been reported to disrupt the pulmonary or endothelial epithelial barrier^30–32^. Nonetheless, the mechanism by which excessive neutrophils injure the gingival epithelial barrier is yet to be clarified. In addition, it was reported that NE inhibitor decreases endotoxemia by improving intestinal permeability and intestinal inflammatory response^33^; however, the positive effect of NE inhibitor on periodontitis has rarely been reported. In this study, NE cleaved cell adhesion molecules and increased gingival epithelial cell monolayer permeability of periodontal pathogen in vitro. Furthermore, the injection of an NE inhibitor downregulated the transcription of proinflammatory cytokine genes in a murine model of periodontitis. These findings indicate that NE leaking from neutrophils aggravates periodontitis by disrupting the gingival epithelial barrier by cleaving cell adhesion molecules and inducing the invasion of periodontal pathogen. Furthermore, NE inhibitors could suppress the damaging effect of NE and eventually prevent aggravation of periodontitis induced by excessive inflammation in vivo.

Our study has some limitations. The injection of an NE inhibitor downregulated the transcription of proinflammatory cytokine genes in the gingiva, however, we could not evaluate the protein levels of proinflammatory cytokines by ELISA. This may be due to low cytokine levels in the gingiva or interference with detection of the cytokines by blood in the gingiva. Therefore, it is unclear whether NE inhibitor truly reduce inflammatory cytokine proteins in the murine model of periodontitis. Another limitation of this study is that Ca9-22 cell, one of the cancer cell lines, were used to determine the disruptive effect of NE on the gingival epithelial barrier. To be precise, the properties of cancer cell lines are different from primary cells. Thus, it is necessary to validate the effect of the NE and NE inhibitor on periodontal epithelial barrier by in vitro study with primary gingival epithelial cells and in vivo study. We also discuss the limitations that the composition of the oral microbiota have not been revealed in this study. Because NE exhibited minimum bactericidal activity against periodontal pathogen, it has the potential to induce compositional changes that causes periodontitis severity. In addition, it was reported that administration of NE inhibitor in septic rats improved gut microbiota composition^34^. The composition of the oral microbiota closely related to the severity of periodontitis^35^, thus, further studies are required to determine the effects of NE and NE inhibitors on the oral microbiota.

In summary, our study demonstrated that the disruptive effect of NE on the gingival epithelial barrier induces bacterial invasion in periodontal deep tissues, aggravating periodontitis. Additionally, NE inhibitor treatment decreased the transcription of proinflammatory cytokine genes and alveolar bone loss in periodontitis. Thus, NE inhibitor application has potential as a local therapeutic strategy for the treatment of periodontitis.

## Materials and methods

### Mice and reagents

The minimum number of male BALB/c mice (45 mice in total) was purchased from Nihon Chemical Co., Ltd. (Tokyo, Japan), maintained under our institutional guidelines, and subjected to experiments at 8 weeks of age. Sterile feed and water were provided ad libitum. All animal experiments were reviewed and approved by the Institutional Animal Care and Use Committee of Niigata University (approval no.: SA00451). In addition, the animal experiments were in accordance with the ARRIVE (Animal Research: Reporting of In Vivo Experiments) guidelines. We confirmed that all experiments were performed in accordance with relevant guidelines and regulations. NE inhibitor, sivelestat, was purchased from Ono Pharmaceutical Co., Ltd (Osaka, Japan) and dissolved in phosphate-buffered saline (PBS) at a concentration of 10 mg/mL. Human NE (Innovative Research, Novi, MI, USA) was diluted to an appropriate concentration with 0.1 M Tris–HCl buffer (pH 8.0) containing 0.5 M NaCl.

### Tooth ligature-induced murine model of periodontitis

A tooth ligature-induced murine model of periodontitis was established as previously described^36^. Briefly, mice randomly divided and anesthetized with 2–3% isoflurane through a nose mask were ligated with a 5-0 silk ligature around the maxillary second molar to induce periodontitis. The sample size (5 mice per group for each experiment) was set as in the previous study^37,38^. Ligation was performed on one mouse from each group in turn to minimize potential confounders. Thereafter, 50 μg NE inhibitor in 5 μL PBS (ligature + NE inhibitor group) or 5 μL PBS was injected into the palatal gingiva of the molar once daily for 3 days for the NE activity assay or 7 days for other analyses. A criterion was set that mice with unraveled ligature will be excluded. No mice were excluded from the study. All animal experiments were conducted that the researcher who analyzed the results was blinded from the actual experiment. The humane endpoint was decided as ≥ 20% reduction in body weight from baseline or signs of intense pain.

### NE activity assay

Three hours after the last injection of NE inhibitor or PBS, the palatal gingival tissues were homogenized in Tris–HCl buffer using BioMasher (Nippi, Tokyo, Japan). These samples were centrifuged at 200 g for 5 min, and NE activity in the supernatant was determined by a method using the NE-specific substrate *N*-methoxysuccinyl-Ala-Ala-Pro-Val p-nitroanilide (Merck Millipore, Billerica, MA, USA), as previously described^39^.

### Measurement of periodontal bone loss

Mouse maxillae were imaged using a stereoscopic microscope (× 20). Periodontal bone loss was calculated according to a previously described method^36^. Briefly, the distance from the cemento-enamel junction to the alveolar bone crest around the ligated second molar was calculated at five points, and the total values were defined as periodontal bone loss (Supplemental Fig. S3).

### Histological analysis

The maxillae prepared from the murine model were fixed in 4% paraformaldehyde solution (FUJIFILM Wako Pure Chemical Corporation, Osaka, Japan) for 24 h. The specimens were decalcified in decalcifying solution B (FUJIFILM Wako Pure Chemical Corporation) for 8 days at 4 °C. The specimens were then embedded in an optimal cutting temperature compound (Sakura Finetek Japan Co., Ltd., Tokyo, Japan) and snap-frozen in liquid nitrogen. The prepared coronal sections (10 μm) were stained with TRAP (FUJIFILM Wako Pure Chemical Corporation) according to the manufacturer’s instructions and then observed using a BIOREVO BZ-9000 microscope (KEYENCE, Osaka, Japan). On the obtained images, all the TRAP-positive multinucleated giant cells in the periodontal ligament of ligated second molar were counted in five random coronal sections of each mouse.

### Immunofluorescence analysis

NE and Ly6G expressions in coronal frozen sections were assessed by immunofluorescence with antibodies specific for NE (Abcam, Cambridge, UK) and Ly6G (Abcam). After overnight incubation with the primary antibody at 4 °C, NE and Ly6G were visualized using Alexa Fluor 488-conjugated anti-rabbit secondary antibody (Invitrogen, Carlsbad, CA) and Alexa Fluor 594-conjugated anti-rat secondary antibody (Invitrogen), respectively. Nuclei were identified using a mounting medium containing 4′,6-diamidino-2-phenylindole (Abcam). The sections were then observed using a confocal laser scanning microscope (Carl Zeiss, Jena, Germany).

### Quantitative real-time polymerase chain reaction (PCR)

RNA was isolated from the palatal gingiva using the TRI Reagent (Molecular Research Center, Inc., Cincinnati, OH, USA) and reverse transcribed using the SuperScript VILO Master Mix (Thermo Fisher Scientific, Waltham, MA, USA). Quantitative PCR was performed using the StepOnePlus real-time PCR system (Thermo Fisher Scientific) according to the manufacturer’s instructions. Values were normalized to those of *Gapdh* mRNA. Relative quantitation was performed using the comparative Ct method. TaqMan probes and primers for *Gapdh, Il6*, *Il1b*, *Tnf*, and *Cxcl1* were obtained from Thermo Fisher Scientific.

### Disruption assay of three-dimensional human oral epithelial tissue models

Three-dimensional human oral epithelial tissue models with the stratum corneum (SkinEthic HEG; Episkin, Lyon, France) were cultured according to the manufacturer’s instructions. Each insert dish with the tissue was transferred to a 24-well plate previously filled with 300 μL of maintenance medium. NE was added to an insert dish at a concentration of 100 mU/mL, followed by incubation at 37 °C in 95% air and 5% CO_2_ for 12 h. Thereafter, the tissue models were carefully removed from the insert dish together with the polycarbonate filter. The tissue models were then embedded in an optimal cutting temperature compound (Sakura Finetek Japan Co., Ltd.) and snap-frozen in liquid nitrogen, and frozen sections (20 μm) were prepared in a cryostat. These sections were stained with hematoxylin–eosin (Sakura Finetek) and imaged using a BIOREVO BZ-9000 microscope (KEYENCE).

### Proteolytic cleavage analysis

Recombinant human (rh) DSG1 (Abcam), rh-occludin (RayBiotech Inc. Norcross, GA, USA), and rh-E-cadherin (Abcam) were treated with various NE concentrations (10–100 mU/mL) at 37 °C for 3 h. Subsequently, samples were separated by SDS-PAGE. DSG1 was detected by western blotting using an anti-DSG1 antibody (Abcam). Occludin and E-cadherin were detected by staining with Coomassie Brilliant Blue (Apro Science, Tokushima, Japan).

### Gingival epithelial cell permeability assay

The human gingival epithelial cell line Ca9-22 was grown using a previously described method^40^. The cells were seeded on the upper compartments of 0.4-μm (for FITC-dextran) or 1-μm (for periodontal pathogen) pore-size Transwell (Corning, NY, USA, and Merck Millipore, respectively) at a density of 1 × 10^5^ cells per well and were cultured for 3 days to form epithelial monolayers. NE was added to the upper compartments at concentrations of up to 100 mU/mL. After 3 h of incubation, the medium in the upper compartments was removed, and 70 kDa of FITC-dextran (100 μg/mL, Cosmo Bio, Tokyo, Japan) or suspensions of *Porphyromonas gingivalis* strain ATCC 33277, *Aggregatibacter actinomycetemcomitans* strain JP2, *Fusobacterium nucleatum* strain ATCC 25586, and *Prevotella intermedia* strain ATCC 25611 were added. After 3 h, the medium was collected from the lower chamber compartments of each group. In the FITC-dextran-added group, the fluorescence intensity of the medium was measured using a GloMax microplate reader (Promega, Madison, WI, USA) at 485-nm excitation and 520-nm emission wavelength. In the bacterial suspension-added group, 100 μL of the medium was plated onto 5% sheep blood agar plates, and bacterial permeability was determined using the colony counting method.

### Statistical analysis

Data were analyzed using GraphPad Prism 7.03 (GraphPad Software, Inc., La Jolla, CA, USA). Significant differences were tested using one-way analysis of variance with Tukey’s multiple comparison method or Dunnett’s multiple comparison method.

## Supplementary Information

Supplementary Figures.

## Data Availability

The datasets generated and analyzed during the current study are available from the corresponding author on reasonable request.
