# Supplementary material for: Neutrophil elastase aggravates periodontitis by disrupting gingival epithelial barrier via cleaving cell adhesion molecules
Source: Sci Rep. 2022 May 17;12:8159. doi: 10.1038/s41598-022-12358-3 (PMC9114116; doi:10.1038/s41598-022-12358-3)

**Supplementary Information**

**Neutrophil elastase aggravates periodontitis by disrupting gingival epithelial barrier via cleaving cell adhesion molecules**

Takumi Hiyoshi^1,2,3^, Hisanori Domon^1,3^, Tomoki Maekawa^1,2,3^, Hikaru Tamura^1,2^, Toshihito Isono^1^, Satoru Hirayama^1^, Karin Sasagawa^1,2^, Fumio Takizawa^1,2^, Koichi Tabeta^2^, Yutaka Terao^1,3*^

^1^Division of Microbiology and Infectious Diseases, Niigata University Graduate School of Medical and Dental Sciences, Niigata, Japan

^2^Division of Periodontology, Niigata University Graduate School of Medical and Dental Sciences, Niigata, Japan

^3^Center for Advanced Oral Science, Niigata University Graduate School of Medical and Dental Sciences, Niigata, Japan

*Corresponding author: Yutaka Terao

Division of Microbiology and Infectious Diseases, Niigata University Graduate School of Medical and Dental Sciences, 2-5274, Gakkocho-dori, Chuo-ku, Niigata-shi, Niigata 951-8514, Japan.

E-mail address: terao@dent.niigata-u.ac.jp

Phone: +81-25-227-2838 / Fax: +81-25-227-0806


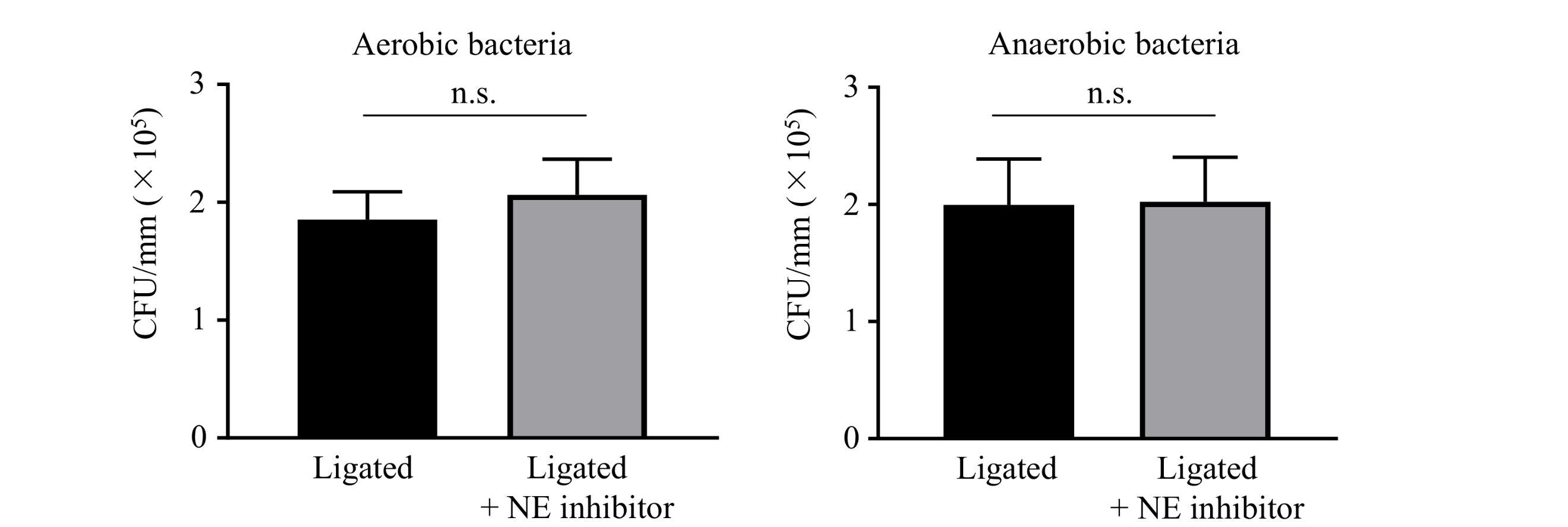
**Supplemental Figure S1. NE inhibitor does not affect bacterial load in ligatures from murine model of periodontitis.**

Male BALB/c mice (Nihon CLEA, Tokyo, Japan) were ligated with a 5-0 silk ligature around the maxillary second molar to induce periodontitis. Thereafter, 5 μL of neutrophil elastase (NE) inhibitor (10 mg/mL, ligature + NE inhibitor group) or PBS (as a control) was injected into the palatal gingiva of the molar once daily for 7 days. The 5-0 silk ligatures sampled from the molars were adjusted to 4 mm and vortexed for 1 min in 1 mL PBS. Serial 10-fold dilutions of the bacterial samples (100 μL) were plated onto 5% sheep blood agar plates (Nippon Becton Dickinson Co., Ltd., Tokyo, Japan). Colony-forming units were evaluated after 1 week of incubation under aerobic or anaerobic conditions to determine the total bacterial counts. An anaerobic jar (Mitsubishi Gas Chemical, Tokyo, Japan) and AnaeroPack™ system (Mitsubishi Gas Chemical) were used to generate anaerobic culture conditions. Data are presented as the mean ± SD (n = 5 per group). The group means were compared using Student’s *t*-test. Colony-forming units, CFU; NE, neutrophil elastase; n.s., not significant PBS, phosphate-buffered saline.


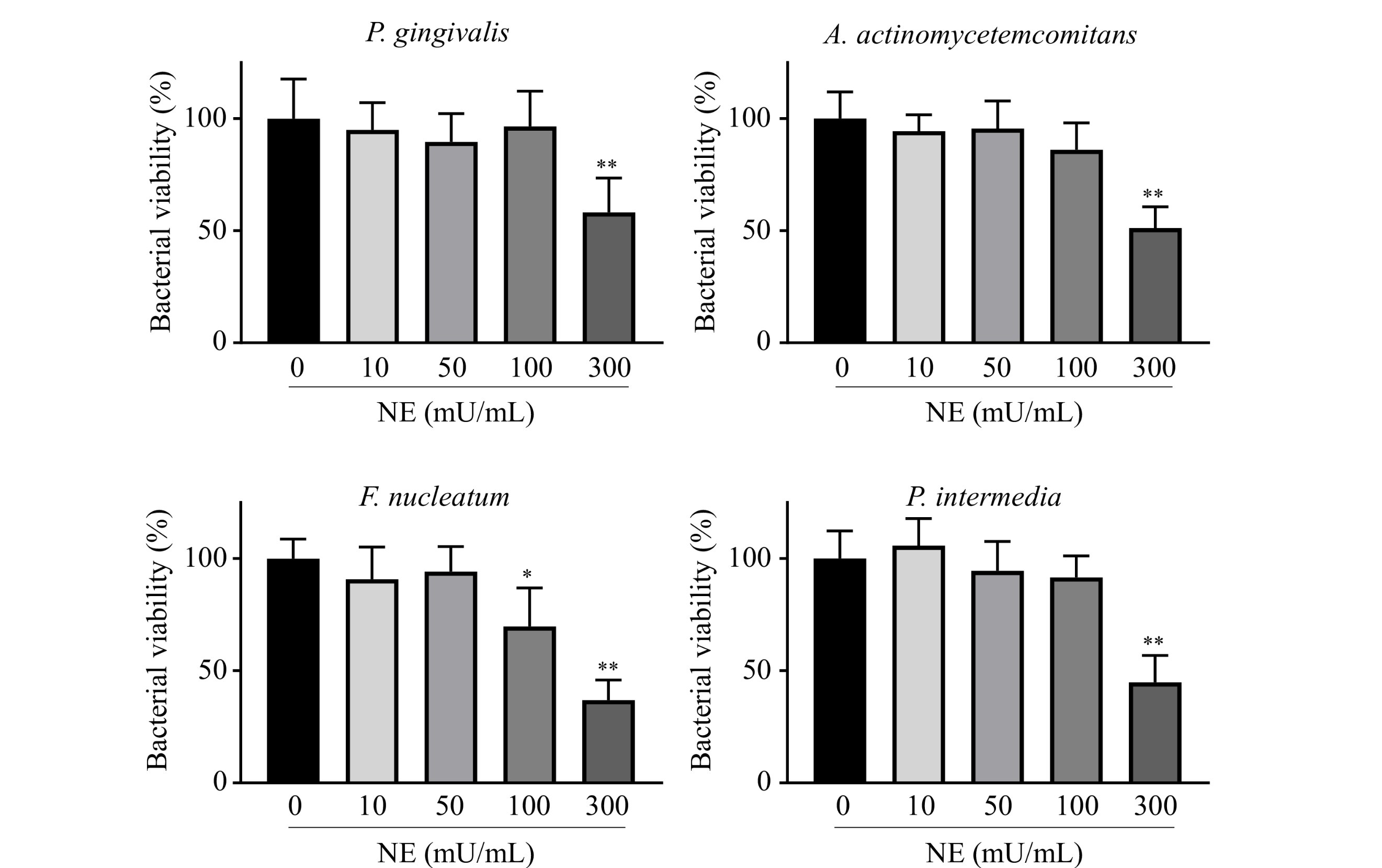
**Supplemental Figure S2.** **NE does not exhibit antibacterial activity up to 100 mU/mL with the exception of *Fusobacterium nucleatum*.**

*Porphyromonas gingivalis* strain ATCC33277, *Aggregatibacter actinomycetemcomitans* strain JP2, *F. nucleatum* strain ATCC25586, and *Prevotella intermedia* strain ATCC25611 were cultured in modified Gifu anaerobic medium broth (Nissui, Tokyo, Japan) under anaerobic conditions at 37°C. All bacteria were centrifuged and diluted in PBS. Thereafter, bacterial suspensions were treated with NE at concentrations of 10–300 mU/mL for 3 h. Serial 10-fold dilutions of the bacterial samples (100 μL) were plated onto 5% sheep blood agar plates and incubated for 1 week. Bacterial viability was determined using the colony counting method. Data are presented as the mean ± SD (n = 5 per group). The group means were compared using one-way analysis of variance with Dunnett’s multiple comparison test. ^*^*p* < 0.05, ^**^*p* < 0.01 compared with the non-NE group. NE, neutrophil elastase.


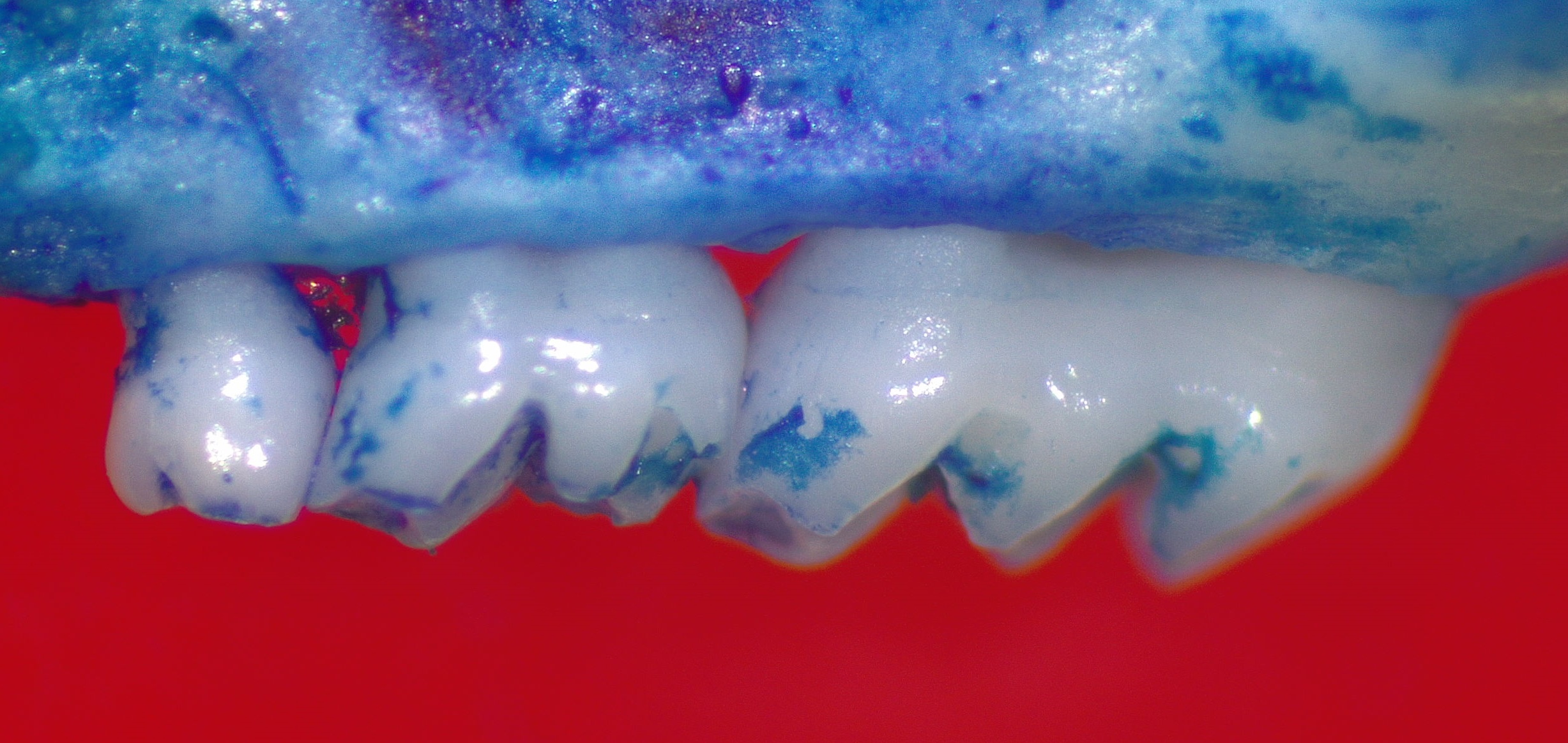


Unligated


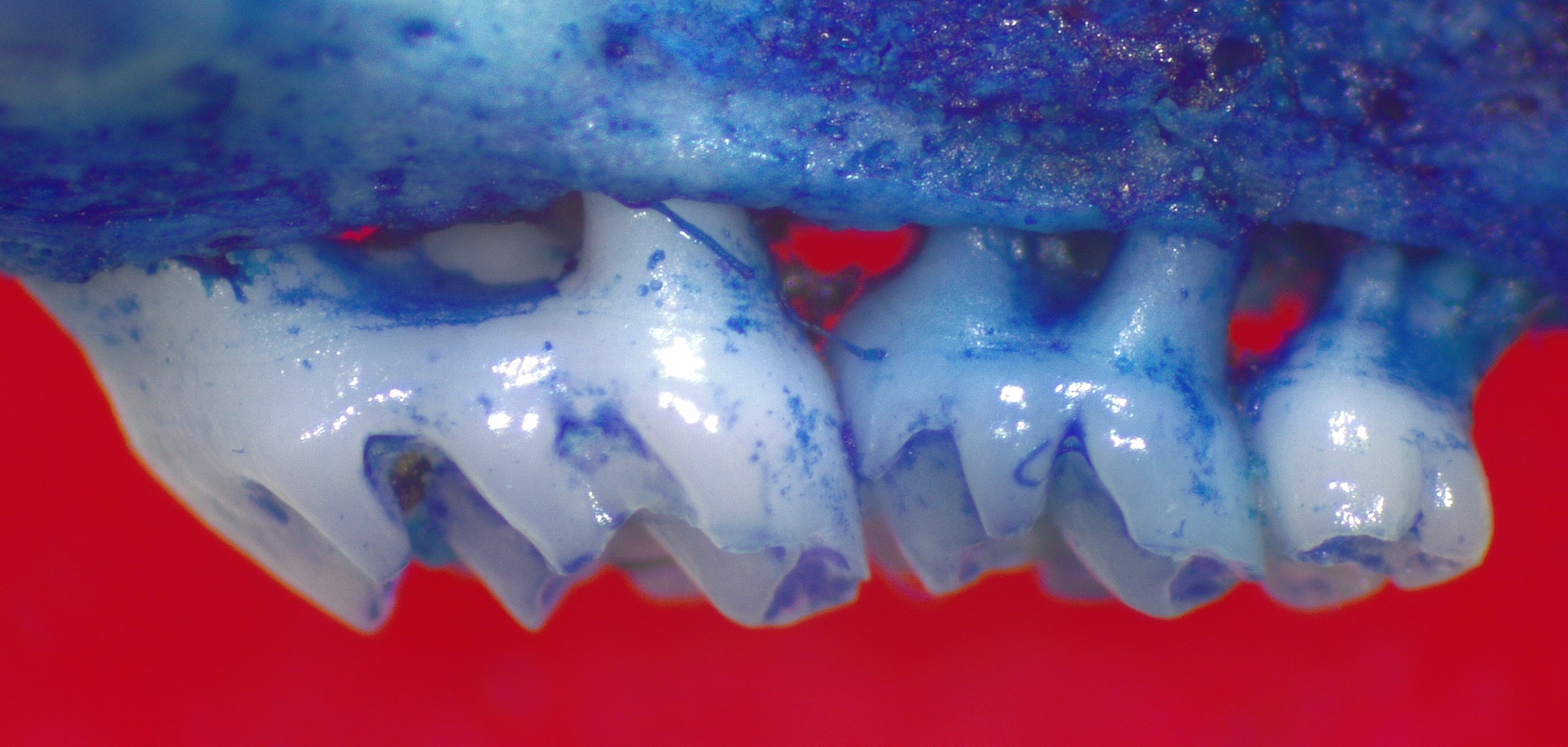


Ligated


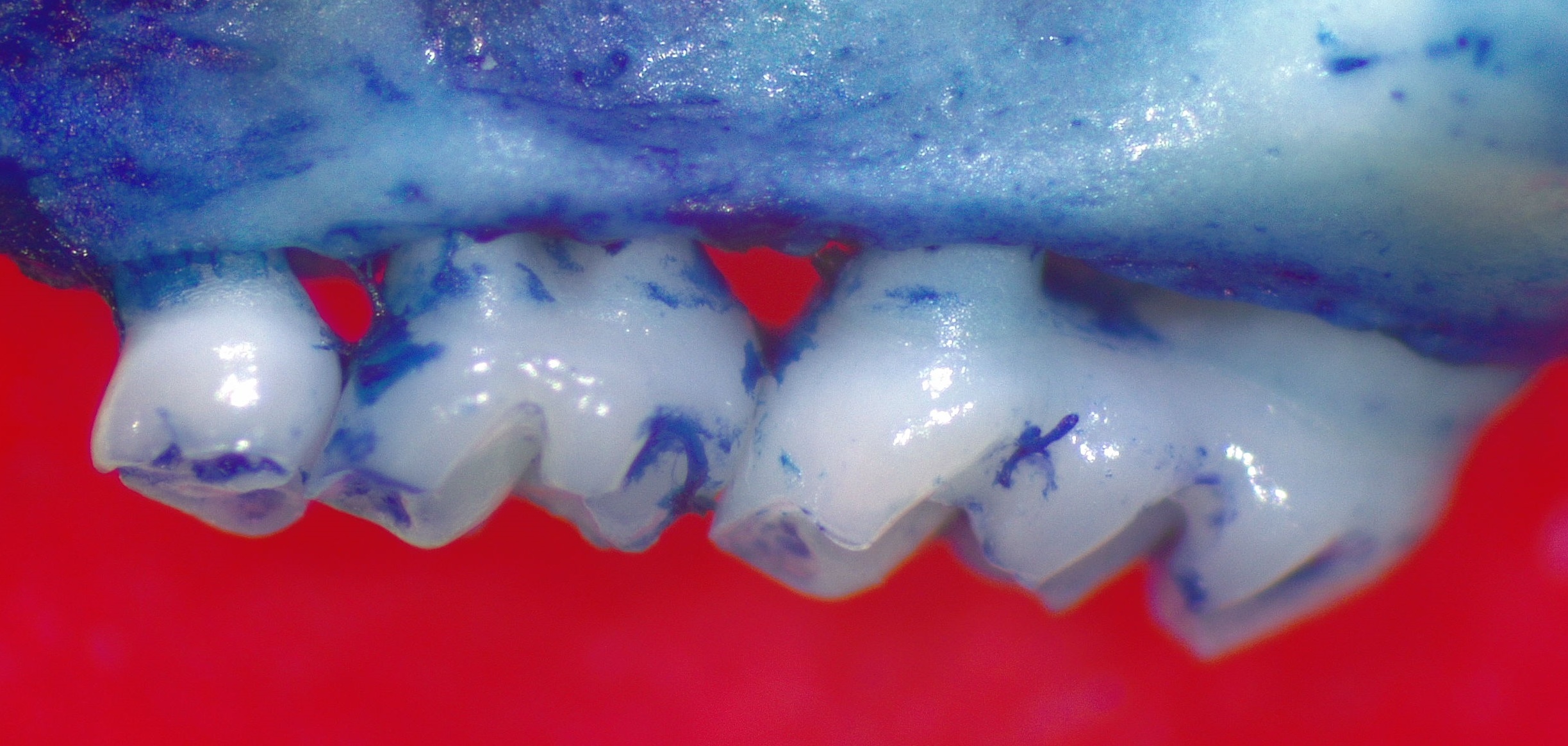


Ligated + NE inhibitor


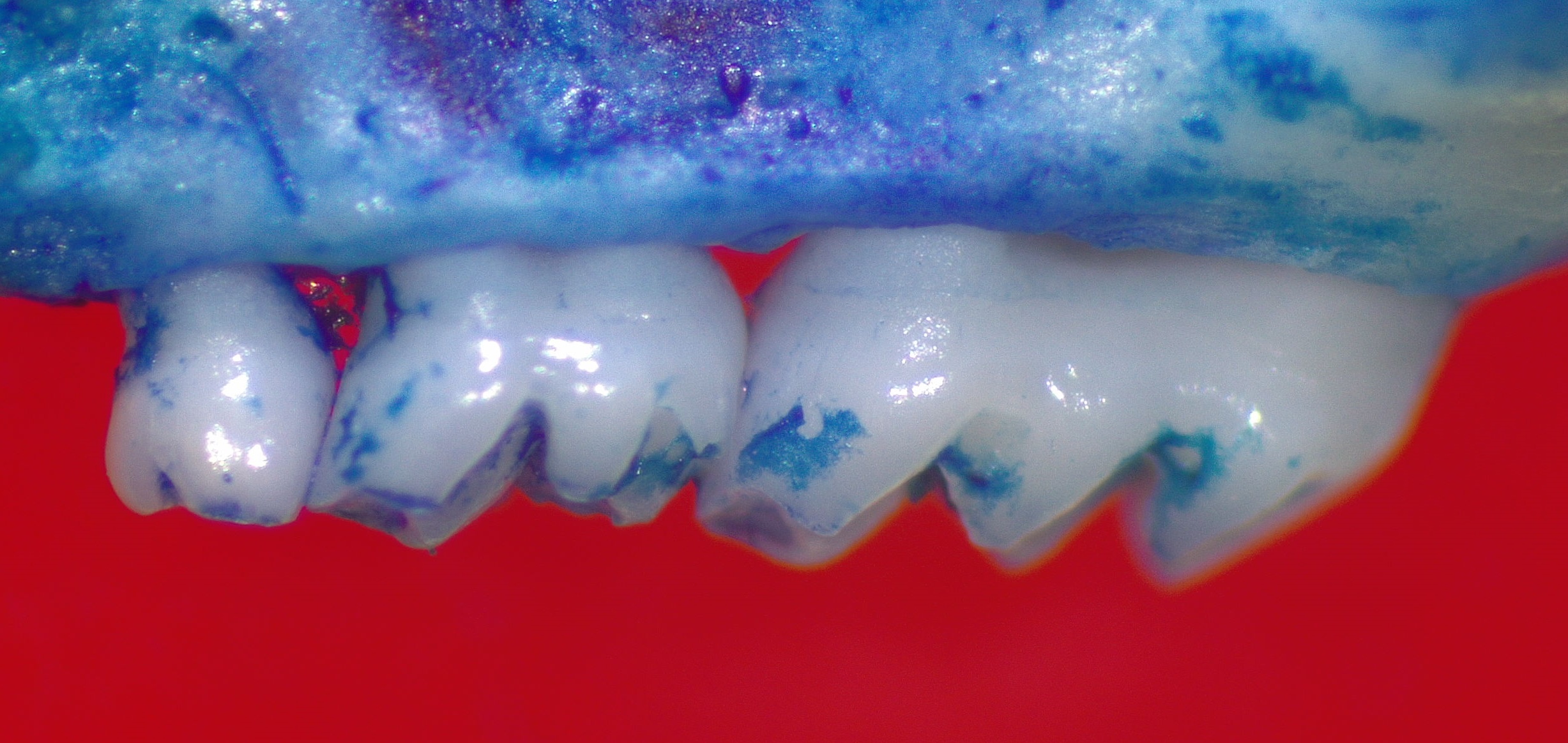


Unligated


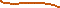

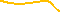

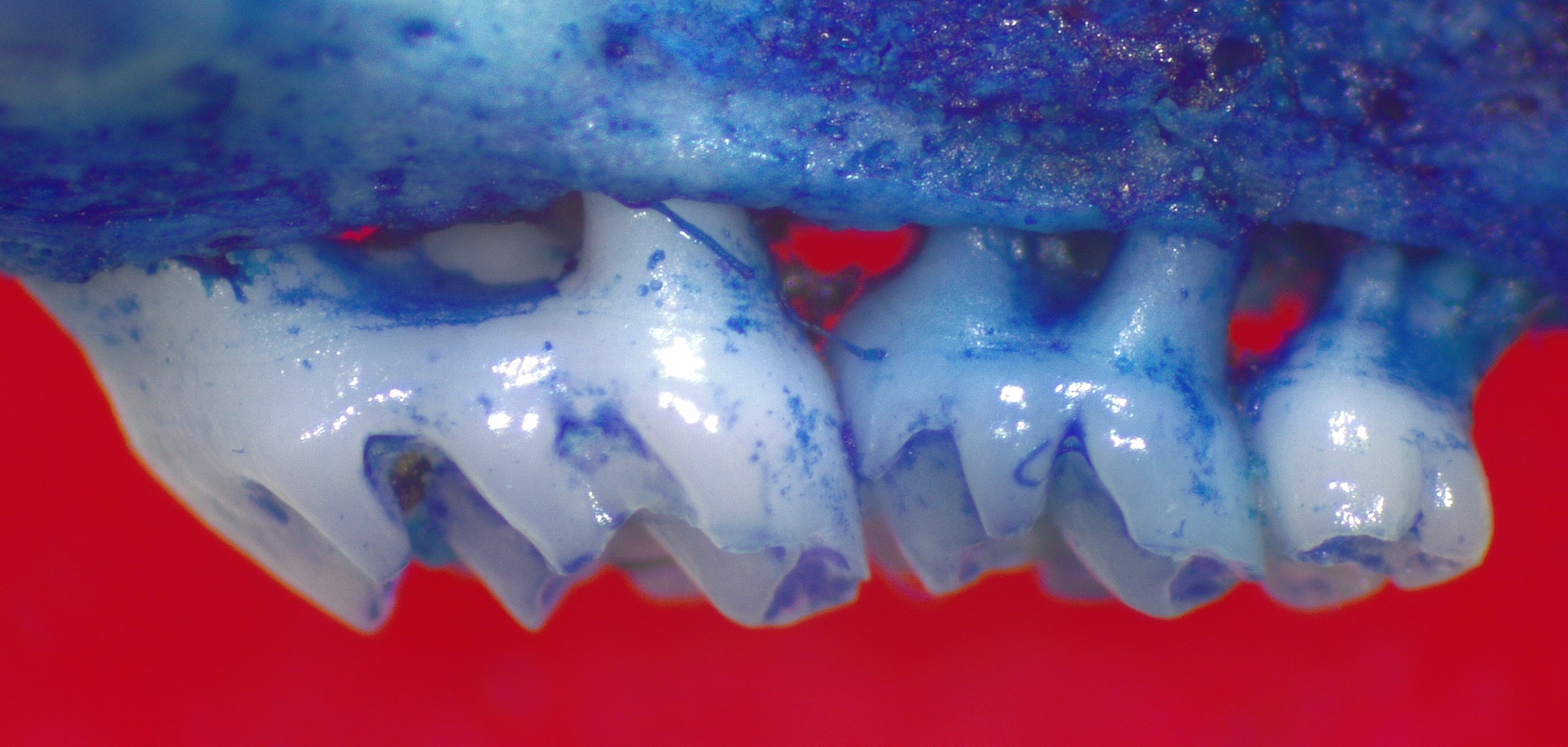


Ligated


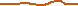

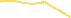

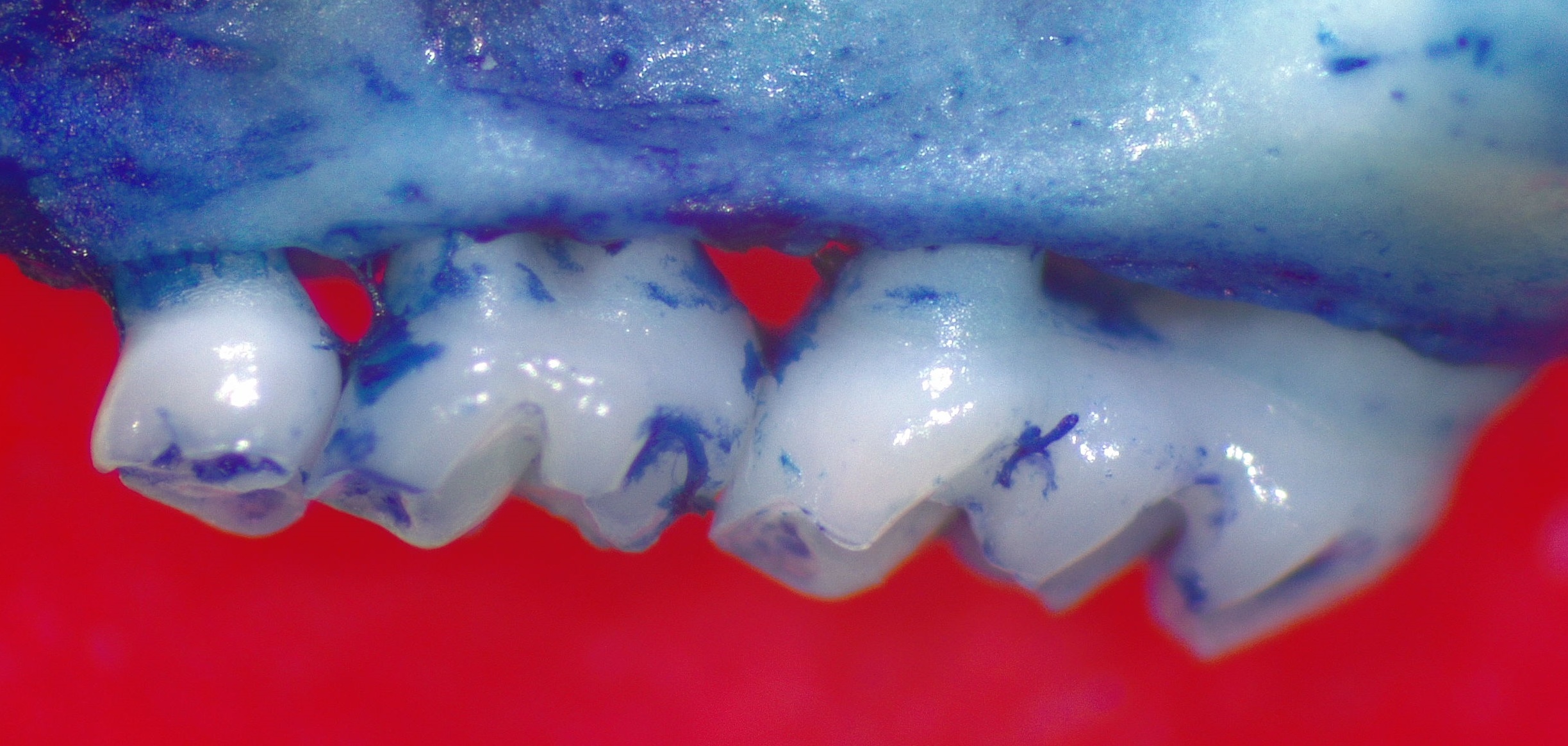


Ligated + NE inhibitor


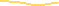

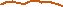


**Supplemental Figure S3. Cemento-enamel junction and the alveolar bone crest** **of the mice maxillae (Figure 2A)**

Yellow lines: cemento-enamel junction

Brown lines: alveolar bone crest

Red: cemento-enamel junction to the alveolar bone crest

NE, neutrophil elastase.

**Original panel for each proteolytic cleavage analysis**

Figure 4C; Desmoglein 1


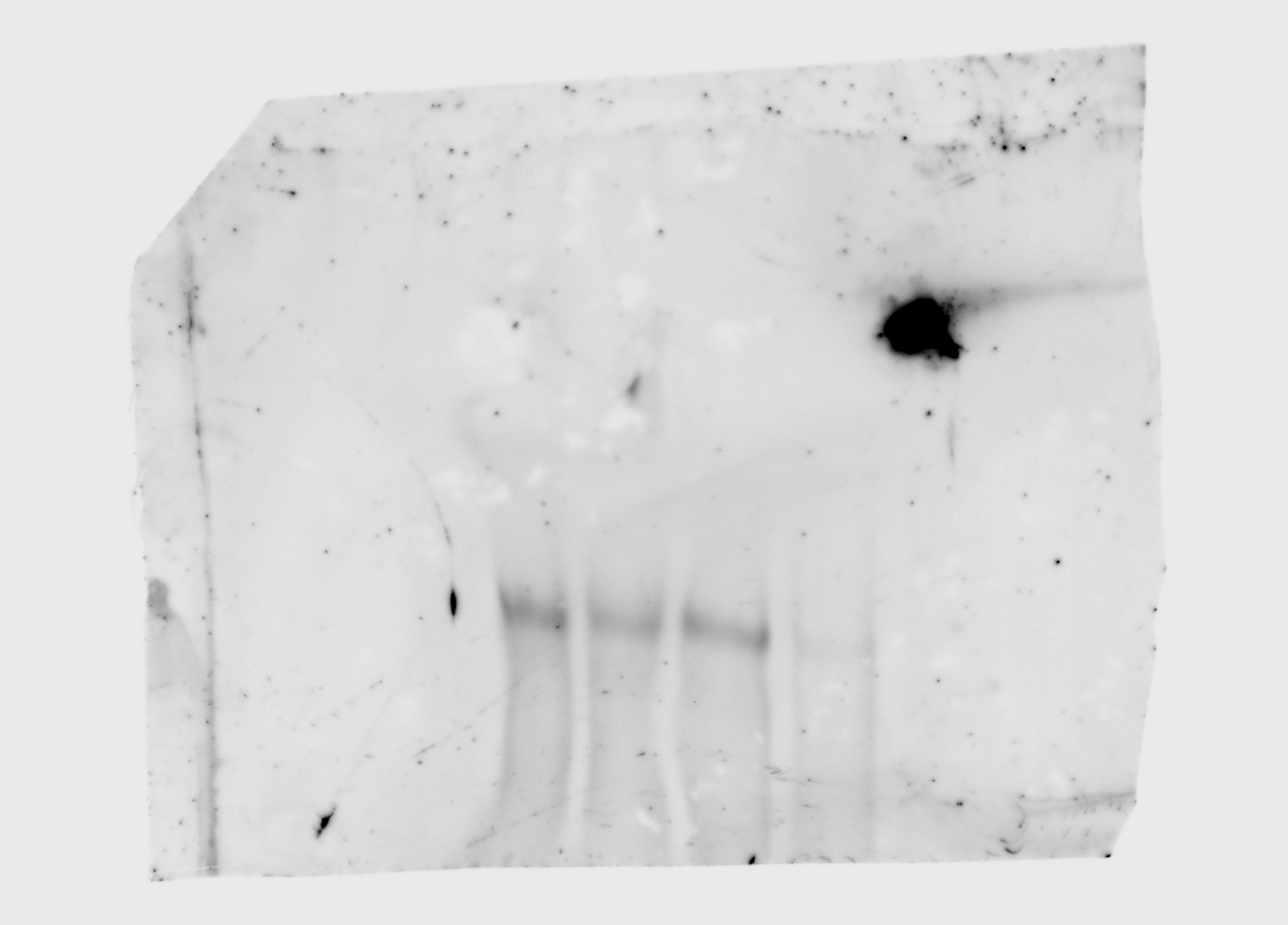


DSG1

Figure 4D; Occludin Figure 4D; E-cadherin


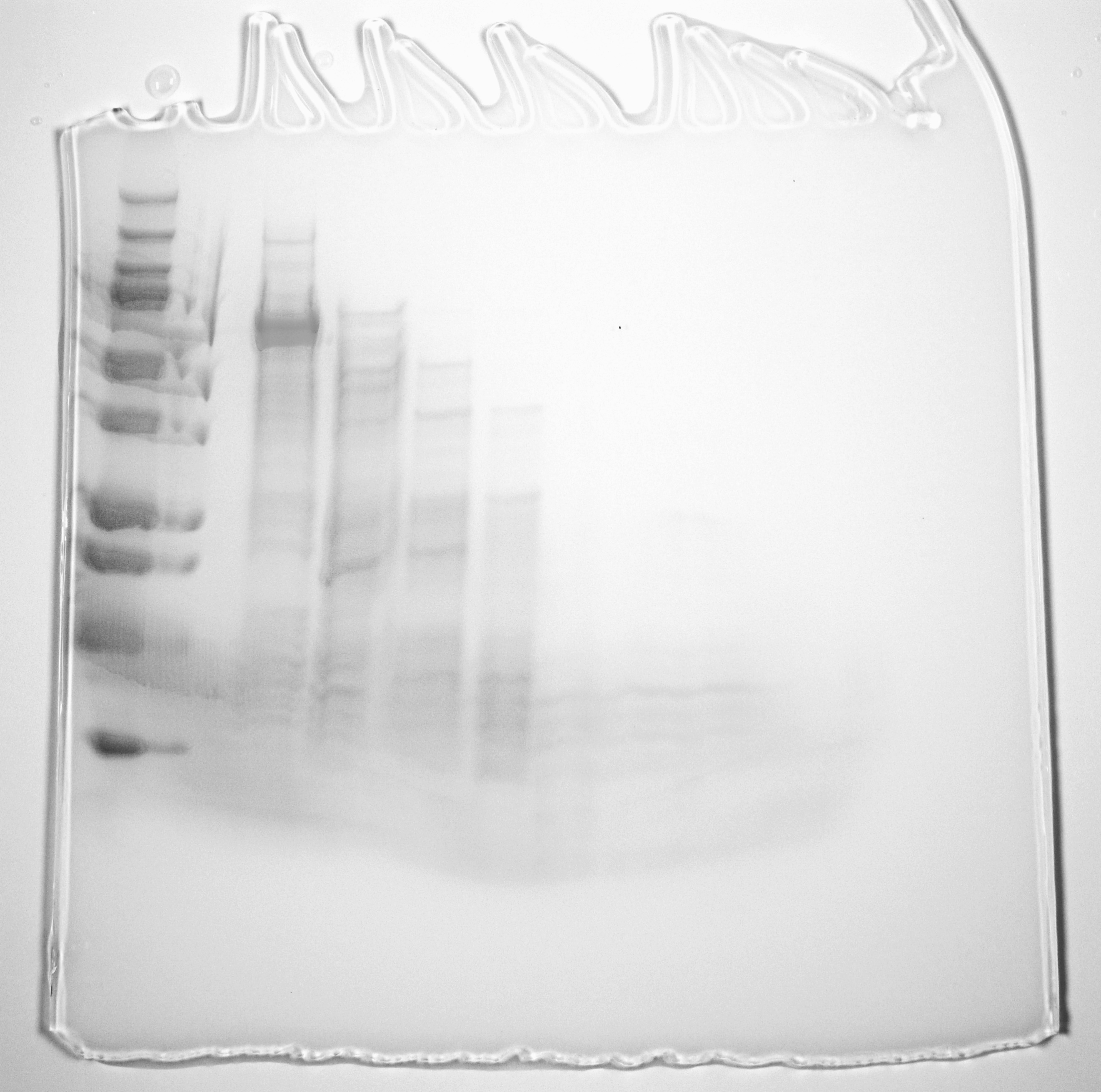

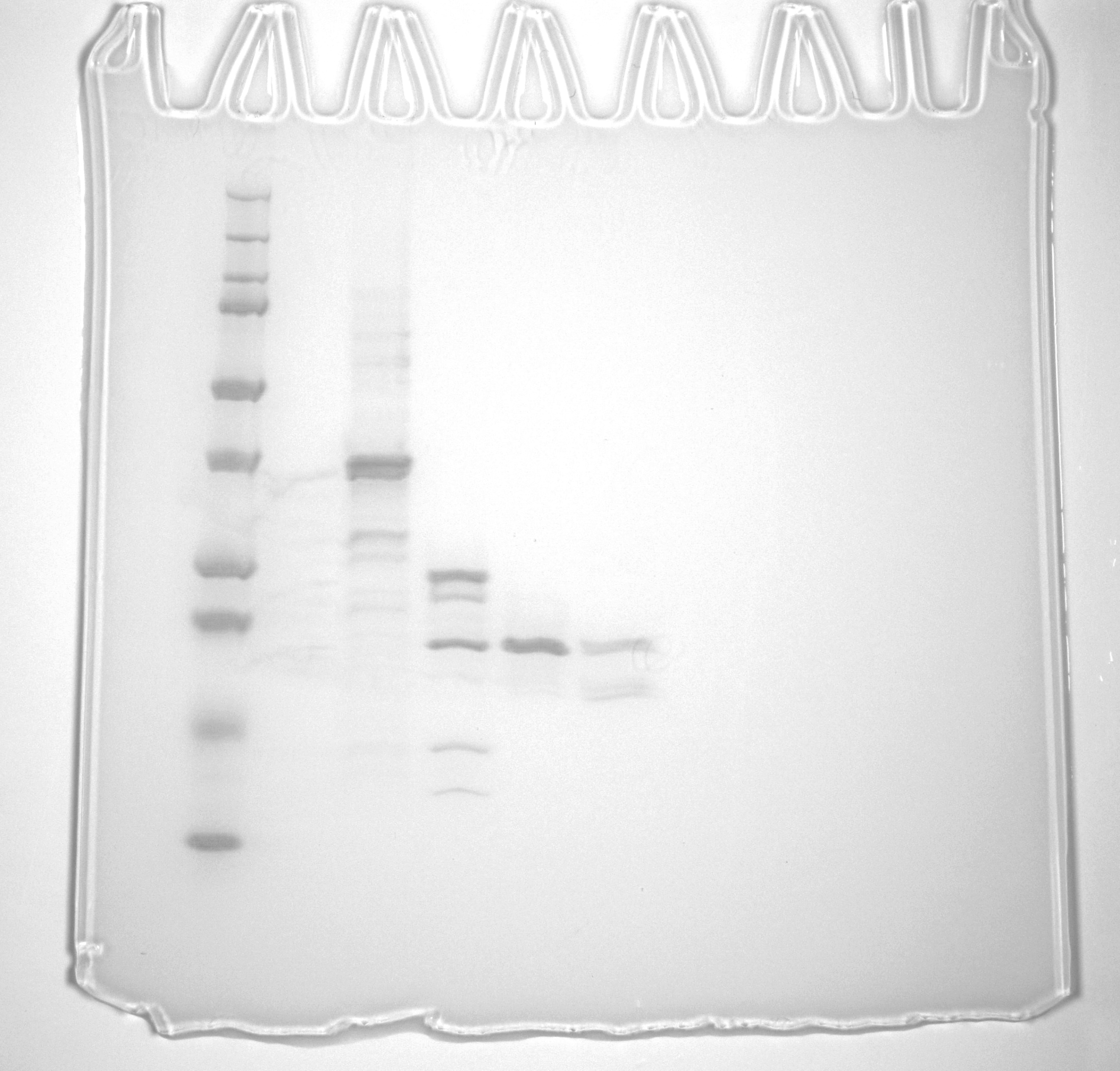

Supplement: Supplementary file 1 — Supplementary Figures. [file 41598_2022_12358_MOESM1_ESM.docx]
